# Extracts from *Annona Muricata* L. and *Annona Reticulata* L. (Annonaceae) Potently and Selectively Inhibit *Plasmodium Falciparum*

**DOI:** 10.3390/medicines2020055

**Published:** 2015-04-30

**Authors:** Lauve Rachel Tchokouaha Yamthe, Patrick Valere Tsouh Fokou, Cedric Derick Jiatsa Mbouna, Rodrigue Keumoe, Bruno Lenta Ndjakou, Paul Toukam Djouonzo, Alvine Ngoutane Mfopa, Jennifer Legac, Nole Tsabang, Jiri Gut, Philip J. Rosenthal, Fabrice Fekam Boyom

**Affiliations:** 1 Laboratory for Phytobiochemistry and Medicinal Plants Studies, Faculty of Science, University of Yaoundé I, P.O. Box 812, Yaoundé, Cameroon; E-Mails: yamthe_lauve@yahoo.fr (L.R.T.Y.); Tsouh80@yahoo.fr (P.V.T.F.); cedrickjiatsa@yahoo.com (C.D.J.M.); rkeumoe@yahoo.fr (R.K.); ngmfalvine@yahoo.fr (A.N.M.); 2 Institute of Medical Research and Medicinal Plants Studies (IMPM), P.O. Box 6163, Yaoundé, Cameroon; E-Mails: touks241@yahoo.fr (P.T.D.); tsabang@hotmail.com (N.T.); 3 Department of Chemistry, Higher Teacher Training College, University of Yaoundé 1. P.O. Box 47, Yaoundé, Cameroon; E-Mail: lentabruno@yahoo.fr; 4 Department of Organic Chemistry, Faculty of Science, University of Yaoundé I, P.O. Box 812, Yaoundé, Cameroon; 5 Division of Infectious Diseases, Department of Medicine, University of California, San Francisco, CA 94943, USA; E-Mails: jlegac@medsfgh.ucsf.edu (J.L.); jiri.gut@ucsf.edu (J.G.); prosenthal@medsfgh.ucsf.edu (P.J.R.)

**Keywords:** *Annona muricata*, *Annona reticulata*, cytotoxicity, *Plasmodium falciparum*, antiplasmodial activity

## Abstract

The aim of this work was to screen extracts from *Annona muricata* and *Annona reticulata in vitro* against *Plasmodium falciparum*. Crude ethanolic extracts, methylene chloride fractions, aqueous fractions, subfractions and isolated compounds (stigmasterol-3-*O*-β-d-glucopyranoside, lichexanthone, gallic acid and β-sitosterol-3-*O*-β-d-glucopyranoside) were tested for cytotoxicity on erythrocytes and Human Foreskin Fibroblasts cells and against the W2 strain of *P. falciparum* in culture. Results indicated that none of the extracts was cytotoxic at concentrations up to 10 µg/mL. Most of the extracts, fractions and subfractions inhibited the growth of *P. falciparum* with IC_50_ values ranging from 0.07 to 3.46 µg/mL. The most potent was the subfraction 30 from *A. muricata* stem bark (IC_50_ = 0.07 µg/mL) with a selectivity index of ˃ 142. Subfraction 3 from *A. muricata* root also exhibited very good activity (IC_50_ = 0.09 µg/mL) with a high selectivity index (SI ˃ 111). Amongst the isolated compounds, only gallic acid showed activity with IC_50_ of 3.32 µg/mL and SI > 10. These results support traditional claims for *A. muricata* and *A. reticulata* in the treatment of malaria. Given their limited cytotoxicity profile, their extracts qualify as promising starting points for antimalarial drug discovery.

## 1. Introduction

Malaria is a life-threatening disease caused by parasites that are transmitted through the bites of infected mosquitoes. According to the latest estimates, there were about 198 million cases of malaria and an estimated 584,000 deaths from malaria in 2013 [1]. Most deaths occur among children living in sub-Saharan Africa, where a child dies from malaria every minute. Malaria mortality rates among children in Africa have been reduced by an estimated 54% since 2000, though it remains one of the most devastating infectious killers [1].

Artemisinin-based combination therapies are now recommended therapies for falciparum malaria in nearly all countries [2]. The recent emergence of artemisinin-resistant parasites in Southeast Asia [3] has highlighted the need for new treatments for malaria.

For centuries, plants have served as a rich source of novel compounds for the treatment of various human diseases. Antimalarial drugs developed from plants include quinine from *Cinchona* tree bark and artemisinin from *Artemisia annua* [4]. Ethnobotanical surveys play an important role in the identification, selection and development of therapeutic agents from medicinal plants. In Cameroon and most parts of Africa, plant extracts are still widely used in the treatment of malaria and several other diseases, in particular in areas where access to standard treatments is limited. However, the potential of many of these plants as sources of antimalarial drugs has yet to be fully explored [5]. A systematic search for Cameroonian plant species with antimalarial activity is underway in our laboratory. Many medicinal plants used for traditional treatment of malaria have been identified, including *Annona muricata* and *Annona reticulata* [5]. *Annona muricata* L*.*, commonly known as graviola or soursop, is a small, upright tropical evergreen tree, 5–6 m high, with large glossy, dark green leaves. It produces a large, heart-shaped, edible fruit that is 15–23 cm in diameter, is yellow–green in color and has flesh inside. All parts of the *A. muricata* tree have been used medicinally in traditional herbal medicine in South America with the following properties and actions: anthelmintic, antiplasmodial, antiparasitic, antimicrobial, antipyretic, sedative, antispasmodic, nervine, hypotensive, anticonvulsant, digestive, antitumor and anticancer [6]. Moreover, aqueous and organic extracts of various organs of *A. muricata* have been previously investigated for antiplasmodial activity [7,8,9,10]. *Annona reticulata* L., commonly known as bullock’s heart, is a low, erect tree with a spreading or rounded crown, and a trunk up to 25–35 cm in diameter. It grows up to 10 m high. The leaves are narrow-lanceolate, alternating, oblong and deciduous, measuring up to 10–20 cm in length and 2–5 cm in width, with conspicuous veins and a bad smell. The fruit measures 8–16 cm in diameter and may be irregular, symmetrically heart-shaped, nearly round, or lopsided, with a depression at the base [11]. In traditional medicines, this plant has various pharmacological activities such as antioxidant, anticancer, analgesic, nervous system depressant, antimalarial, anthelmintic, and anti-syphilitic [11].

This report describes the *in vitro* antiplasmodial activity of extracts from two Annonaceae, *A. muricata* and *A. reticulata*, which are traditionally used to treat malaria in Cameroon and elsewhere.

## 2. Experimental Section

### 2.1. Plant Collection and Authentication

Plants were selected based on the results of an ethnopharmacological survey on Annonaceae species used traditionally to control malaria and fever in Cameroon [5].

Plants were collected at the University of Yaoundé 1 Campus and Shell Nsimeyong in Yaoundé, Cameroon in May 2011 and July 2013 for *A. muricata* and *A. reticulata*, respectively. Voucher specimens of plant samples were deposited under the respective reference numbers 32879/HNC and 66886/HNC. Collected samples were air dried and ground before extraction.

### 2.2. Preparation of Extracts and Fractionation

The extracts were prepared following the method previously described [12] that was designed to prepare acetogenin-rich fractions, with slight modifications. The materials were submitted to a 95% ethanol extraction for 48 h to yield the ethanolic crude extracts. The ethanolic extracts were partitioned between H_2_O and CH_2_Cl_2_. The CH_2_Cl_2_ layers were submitted to vacuum evaporation using a rotating evaporator (BUCHI 011). H_2_O layers were dried under ventilation at room temperature. Crude ethanolic extracts, CH_2_Cl_2_ fractions and H_2_O fractions were tested for antiplasmodial activity.

Further fractionation and antiplasmodial screening were performed on the CH_2_Cl_2_ fractions of root and stem bark extracts of *A. muricata*.

The CH_2_Cl_2_ fraction of the root extract of *A. muricata* (31.67 g) was subjected to column chromatography over silica gel (Merck, 230–400 mesh) and eluted with hexane, hexane/EtOAc, EtOAc, EtOAc/MeOH and MeOH, in increasing polarity. One hundred and forty four fractions of 250 mL each were collected and subsequently combined according to their TLC profiles on a pre-coated silica gel 60 F plate developed with n-hexane/EtOAc and CH_2_Cl_2_/MeOH mixtures, to give eight subfractions (AMrSF1-AMrSF8) and one compound (AMrP1).

Similarly, column chromatography of 51.75 g of the CH_2_Cl_2_ fraction of the stem bark extract of *A. muricata* led to 388 fractions subsequently combined into 43 subfractions (AMsbSF1-AMsbSF43) and three products, codified AMsbP1, AMsbP2 and AMsbP3.

The structures of isolated products were elucidated using spectroscopic analysis (proton- and carbon 13-NMR, mass spectra (MS), COSY, HSQC and HMBC) and confirmed by comparison with published results.

### 2.3. Evaluation of Biological Activities

#### 2.3.1. Erythrocyte Susceptibility to Plant Extracts

A preliminary toxicological assessment was carried out to determine the highest drug concentrations that could be incubated with erythrocytes without apparent toxicity. This was done according to the 3-[4,5-dimethylthiazol-2-yl]-2,5-diphenyltetrazolium bromide/phenazine methosulfate (MTT/PMS, Promega) colorimetric assay [13], with some modifications [14]. This method is based on the reduction of MTT to formazan by enzymes of viable cells, especially the dehydrogenases such as glucose-6-phosphate dehydrogenase of the pentose phosphate pathway of erythrocytes. The extract stock solutions in 10% DMSO (1 mg/mL) were serially diluted in 96 well culture plates using RPMI 1640 and tested at the highest concentration of 20 μg/mL in triplicate against erythrocytes (2% hematocrit) (at 37 °C, in a 3% O_2_, 5% CO_2_ and 91% N_2_ atmosphere, in the presence of RPMI 1640, 25 mM HEPES, pH 7.4 for 48 h). At the end of the incubation period, the cultures were transferred into polypropylene microcentrifuge tubes and centrifuged at 1500 rpm for 5 min, and the supernatant was discarded. A total of 1.5 mL MTT solution with 250 μL g PMS was added to the pellets. Controls contained no erythrocytes. The tubes were thereafter incubated for 45 min at 37 °C, and then centrifuged, and the supernatant was discarded. The pellets were re-suspended in 0.75 mL of HCl 0.04 M in isopropanol to extract and dissolve the dye (formazan) from the cells. After 5 min, the tubes were vigorously mixed and centrifuged, and the absorbance of the supernatant was determined at 570 nm, with absorbance representing healthy cells. The highest drug concentrations producing minimal damage to the cells were considered starting points for drug dilutions.

#### 2.3.2. Human Foreskin Fibroblast (HFF) Susceptibility to Plant Extracts

To determine selectivity indices of active extracts, a toxicological assessment was carried out with human foreskin fibroblast (HFFs), essentially as previously described [15]. Briefly, serially diluted extracts in 2% DMSO were incubated with HFF cells (ATCC-HS68) in culture using a 96-well plate format (Costar). Negative controls consisted of cells without inhibitor. Cultures were incubated for 24 h at 37 °C in humidified CO_2_, and 20 µL of MTS/PMS (Promega, Madison, USA) was added to each well and incubated for 1.5 h at 37 °C. Absorbance was then recorded at 490 nm using a 96-well plate reader (Biotek EL800, Vermont, NE, USA). The percent growth inhibition was calculated from the optical densities relative to the negative control, and 50% cell cytotoxicity (CC_50_) values were determined using GraphPad Prism 5.0. Selectivity indices of plants extracts, defined as the ratio CC_50_ /IC_50_ parasites, were determined.

#### 2.3.3. Antiplasmodial Activity

*Plasmodium falciparum* strain W2 was maintained in culture in sealed flasks at 37 °C, in a 3% O_2_, 5% CO_2_ and 91% N_2_ atmosphere in RPMI 1640, 25 mM HEPES, pH 7.4, supplemented with heat inactivated 10% human serum and human erythrocytes to achieve a 2% hematocrit. Parasites were synchronized at the ring stage by serial treatment with 5% sorbitol (Sigma, Taufkirchen, Germany) [16] and studied at 1% parasitemia. Plant extracts were prepared as 1 mg/mL stock solutions in dimethyl sulfoxide (DMSO), further diluted as needed for individual experiments, and tested in triplicate. The stock solutions were diluted in supplemented RPMI 1640 medium so as to have at most 0.1% DMSO in the final reaction medium. An equal volume of 1% parasitemia, 4% hematocrit culture was thereafter added and gently mixed thoroughly. Negative controls contained equal concentrations of DMSO. Positive controls contained artemisinin (Sigma, Taufkirchen, Germany). Cultures were incubated at 37 °C for 48 h. Parasites at the ring stage were thereafter fixed by replacing the serum medium by an equal volume of 1% formaldehyde in PBS. Aliquots (50 µL) of each culture were then added to 5 mL round-bottom polystyrene tubes containing 0.5 mL 0.1% Triton X-100 and 1 nM YOYO nuclear dye (Molecular Probes) in PBS, and parasitemias of treated and control cultures were compared using a Becton-Dickinson FACSort flow cytometer to count nucleated (parasitized) erythrocytes. Data acquisition was performed using CellQuest software. These data were normalized to percent control activity and IC_50_s were calculated using Prism 5.0 software (GraphPad, CA, USA) with data fitted by non-linear regression to the variable slope sigmoidal dose–response formula, *y* = 100/1 + 10^(log^^IC50−*x*)H^, where H is the hill coefficient or slope factor [17].

## 3. Results and Discussion

### 3.1. Plant Extraction and Fractionation

**Figure 1:**
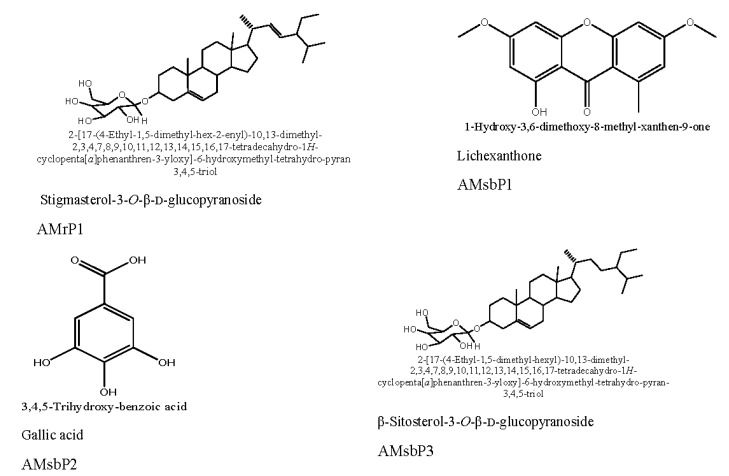
Chemical structures of compounds isolated from *A*. *muricata*.

From each investigated plant organ, an ethanolic crude extract was prepared and subsequently partitioned into H_2_O and CH_2_Cl_2_ fractions. They were all tested for biological activity, and the CH_2_Cl_2_ fractions of *A. muricata* root and stem bark (AMrCH_2_Cl_2_, AMsbCH_2_Cl_2_) were selected as promising (Table 1) and subjected to flash chromatography as described in the materials and methods section to afford the chemically known constituents AMrP1, AMsbP1, AMsbP2 and AMsbP3 (Figure 1).

Isolated compounds were identified by comparison of their spectroscopic data with those reported in the literature as stigmasterol-3-*O*-β-d-glucopyranoside (AMrP1) [18,19], lichexanthone (AMsbP1) [20,21], gallic acid (AMsbP2) [22,23] and β-sitosterol 3-*O*-β-d-glucopyranoside (AMsbP3) [24]. Stigmasterol-3-*O*-β-d-glucopyranoside (AMrP1) was reported in the root of *Polyalthia* *longifolia* var pendula [25]. Lichexanthone (AMsbP1) had previously been isolated from the root of *Rollinia leptopetala* [26] and from the bark of *Guatteria blepharophylla* [27]. Gallic acid (AMsbP2) and β-sitosterol 3-*O*-β-d-glucopyranoside (AMsbP3) were recently found in the leaf of *Polyalthia longifolia* [28] and twig of *Annona squamosa* [29] respectively, all plants belonging to the Annonaceae family.

The 79 extract samples prepared from the two investigated plants were screened for antiplasmodial activity.

Crude extracts from the pericarp, root, and stem bark of *A. muricata* and stem bark, root, and fruit of *A. reticulata* showed antiplasmodial activity, with IC_50_ values ranging from 0.29 to 1.90 µg/mL (Table 1). Crude ethanolic extracts from the leaf and twig of *A. reticulata* were inactive (IC_50_ > 10 µg/mL).

From the crude ethanolic extracts, CH_2_Cl_2_ and H_2_O fractions were prepared by liquid-liquid partitions. Overall, none of the H_2_O fractions showed activity. On the other hand, apart from the ARlCH_2_Cl_2_ fraction from *A. reticulata* leaf, the seven other CH_2_Cl_2_ fractions from both plants exhibited potent activity, with IC_50_ values ranging from 0.19–1.50 µg/mL (Table 1). The most potent was from the root extract (AMrCH_2_Cl_2_-IC_50_ = 0.19 µg/mL). Overall, the fractions AMpCH_2_Cl_2_, AMrCH_2_Cl_2_, AMsbCH2Cl_2_, ARtwCH_2_Cl_2_, AResbCH_2_Cl_2_, ARrCH_2_Cl_2_ and ARfrCH_2_Cl_2_ were promising, with IC_50_ values below 5 µg/mL. Fraction AMrCH_2_Cl_2_ showed the best selectivity (SI ˃ 52.6).

**Table 1:** Susceptibility of human foreskin fibroblast (HFF) and *P. falciparum* to plant extracts.

Plant Species	Organ	Nature of Extract	Code	^a^ Yield (%)	^b^ IC_50_ (µg/mL) ± S.D.	^c^ SI
*Annona muricata*	Pericarp	Crude ethanol extract	AMpEthOH	5.68	1.01 ± 0.07	˃9.90
		H_2_O fraction	AMpH_2_O	1.02	>10	ND
		CH_2_Cl_2_ fraction	AMpCH_2_Cl_2_	3.54	0.94 ± 0.03	˃10.63
	Root	Crude ethanolic extract	AMrEthOH	6.23	0.79 ± 0.14	˃12.65
		H_2_O fraction	AMrH_2_O	0.98	>10	ND
		CH_2_Cl_2_ fraction	AMrCH_2_Cl_2_	4.18	0.19 ± 0.03	˃52.63
		Subfractions	AMrSF1	0.56	0.61 ± 0.04	˃16.39
			AMr SF2	0.33	0.22 ± 0.06	˃45.45
			AMrSF3	1.07	0.09 ± 0.003	˃111.11
		Purified compound	Stigmasterol-3-*O*-β-d-glucopyranoside (AMrP1)	0.016	˃10	ND
	Stem bark	Crude ethanolic extract	AMsb-EthOH	5.91	1.45 ± 0.20	˃6.45
		H_2_O fraction	AMsbH_2_O	0.87	>10	ND
		CH_2_Cl_2_ fraction	AMsbCH_2_Cl_2_	4.32	1.50 ± 0.07	˃6.66
		Subfractions	AMsbSF2	0.11	1.65 ± 1.58	˃6.06
			AMsbSF15	0.08	2.52 ± 1.41	˃3.96
			AMsbSF16	0.16	3.46 ± 0.98	˃2.89
			AMsbSF17	0.04	2.45 ± 1.77	˃3.92
			AMsbSF18	0.02	2.75 ± 1.86	˃3.63
			AMsbSF19	0.73	2.89 ± 0.55	˃3.46
			AMsbSF20	0.03	1.14 ± 0.22	˃8.77
			AMsbSF21	0.03	1.11 ± 0.34	˃9.00
			AMsbSF22	0.02	0.12 ± 0.03	˃83.3
			AMsbSF24	0.41	0.46 ± 0.04	˃21.7
			AMsbSF27	0.02	1.17 ± 0.16	˃8.54
			AMsbSF28	0.62	0.72 ± 0.07	˃13.88
			AMsbSF29	0.07	0.75 ± 0.13	˃13.33
			AMsbSF30	0.31	0.07 ± 0.009	˃142.3
			AMsbSF31	0.19	0.28 ± 0.05	˃35.7
			AMsbSF32	0.017	0.78 ± 0.43	˃12.8
			AMsbSF33	0.05	1.31 ± 0.19	˃7.63
			AMsbSF34	0.09	1.07 ± 0.36	˃9.34
			AMsbSF35	0.019	2.19 ± 0.47	˃4.56
			AMsbSF36	0.22	1.39 ± 0.33	˃7.19
		Purified compounds	Lichexanthone (AMsbP1)	0.014	˃10	ND
			Gallic acid (AMsbP2)	0.052	3.32 ± 1.49	˃3.01
			β-Sitosterol-3-O-β-D-glucopyranoside (AMsbP3)	0.012	˃10	ND
*Annona reticulata*	Leaf	Crude ethanolic extract	ARlEthOH	10.46	˃ 10	ND
		H_2_O fraction	ARlH_2_O	1.71	>10	ND
		CH_2_Cl_2_ fractions	ARlCH_2_Cl_2_	9.93	˃ 10	ND
	Twig	Crude ethanolic extract	ARtwEthOH	4.96	˃ 10	ND
		H_2_O fraction	ARtwH_2_O	0.53	>10	ND
		CH_2_Cl_2_ fraction	ARtwCH_2_Cl_2_	1.78	0.88 ± 0.34	˃11.4
	Stem bark	Crude ethanolic extract	ARsbEthOH	6.02	0.29 ± 0.02	˃34.5
		H_2_O fraction	ARsbH_2_O	1.04	>10	ND
		CH_2_Cl_2_ fraction	ARsbCH_2_Cl_2_	3.71	0.82 ± 0.25	˃12.2
	Root	Crude ethanolic extract	ARrEthOH	5.12	1.90 ± 0.008	˃5.26
		H_2_O fraction	ARrH_2_O	0.71	>10	ND
		CH_2_Cl_2_ fraction	ARrCH_2_Cl_2_	4.54	0.38 ± 0.23	˃26.3
	Fruit	Crude ethanolic extract	ARfrEthOH	5.14	0.67 ± 0.02	˃14.9
		H_2_O fraction	ARfrH_2_O	0.48	>10	ND
		CH_2_Cl_2_ fraction	ARfrCH_2_Cl_2_	2.12	0.42 ± 0.009	˃23.8
Positive control			Artemisinin		0.005 ± 0.0008	ND

^a^ The percent extraction yields were calculated in percentages (w/w); The susceptibility of HFF cells to plant samples was evaluated in culture; ^b^ The susceptibility of the W2 strain of *P. falciparum* to plant extracts was evaluated in culture; ^c^ SI = Selectivity index; AM = *Annona muricata*; AR = *Annona reticulata*; CC_50_ = concentration of extract that killed 50% of HFF cells, relative to negative control;IC_50_ = concentration of extract that killed 50% of parasites, relative to negative control; S.D. = standard deviation; ND = not determined.

### 3.2. Susceptibility of Erythrocytes, HFF Cells, and P. Falciparum to Plant Extracts

All the tested extracts showed no toxicity to erythrocytes and HFF cells at concentrations up to10 μg/mL, indicating IC_50_ values greater than 10 μg/mL.

Further fractionation coupled with biological screening was performed on the two CH_2_Cl_2_ fractions of root (AMrCH_2_Cl_2_) and stem bark (AMsbCH_2_Cl_2_) of *A. muricata*. They were submitted to flash chromatography eluting with solvent systems of increasing polarity (Hex-EtOAC 100:0-0:100, and EtOAC-MeOH 95-5–0:100) to afford subfractions and four pure compounds (stigmasterol-3-*O*-β-d-glucopyranoside (AMrP1), lichexanthone (AMsbP1), gallic acid (AMsbp2) and β-sitosterol-3-*O*-β-d-glucopyranoside (AMsbP3)) that were assessed for antiplasmodial activity. Three of eight subfractions (AMrSF1, AMrSF2 and AMrSF3) obtained from the root extract of *A. muricata* possessed activity, with IC_50_ values of 0.61 µg/mL, 0.22 µg/mL and 0.09 µg/mL, respectively (Table 1), with improved activity compared to the initial crude ethanolic extract (AMrEthOH-IC_50_ = 0.79 µg/mL). The isolated compound from the root of *A. muricata*, stigmasterol-3-*O*-β-d-glucopyranoside, did not show activity at concentrations up to 10 µg/mL.

The CH_2_Cl_2_ fraction of *A. muricata* stem bark extract yielded 43 subfractions among which 18 (AMsbSF2, AMsbSF15, AMsbSF16, AMsbSF17, AMsbSF18, AMsbSF19, AMsbSF20, AMsbSF21, AMsbSF22, AMsbSF24, AMsbSF27, AMsbSF28 AMsbSF29, AMsbSF30, AMsbSF31, AMsbSF32, AMsbSF33, AMsbSF34, AMsbSF35 and AMsbSF36) showed antiplasmodial activity (IC_50_ < 5 µg/mL), with IC_50_ values ranging from 0.07 µg/mL (AMsbSF30) to 3.46 µg/mL (AMsbSF16). Among the three isolated compounds from this CH_2_Cl_2_ fraction (lichexanthone, gallic acid and β-sitosterol 3-*O*-β-d-glucopyranoside), only gallic acid showed activity, with IC_50_ 3.32 µg/mL. Subfraction AMsbSF30 exerted the best activity (IC_50_ = 0.07 µg/mL) and also showed the highest selectivity (SI = 142.3).

Moreover, according to previously agreed criteria [30], Subfraction 3 of the root extract (AMrSF3) and Subfraction 29 of the stem bark extract (AMsbSF30) from *A. muricata* that showed IC_50_ values below 0.2 µg/mL and SI ˃ 100 could be considered as promising starting points for drug development. Furthermore, SI ˃ 10 indicates appropriate pharmacological efficacy and safety of plant extracts [31]. Based on these criteria, 19 samples out of the 79 extracts, fractions, and subfractions tested showed acceptable selectivity (SI ˃ 10.6–142.3).

In our previous report [5], Annonaceae used to treat malaria, including *A. muricata*, were described. The use of *A. reticulata* in the traditional cure of malaria was reported [11]. In the present study, extracts from *A. muricata* and *A. reticulata* showed antiplasmodial activity, with IC_50_ values < 10 µg/mL, and did not demonstrate cytotoxicity.

Extracts from *A. muricata* pericarp, root and stem bark showed potent antiplasmodial activity. Results obtained with *A. muricata* crude ethanolic extracts from pericarp, root and stem bark and their respective CH_2_Cl_2_ fractions corroborated the findings of other authors [7]. Twenty micrograms per milliliter of soaked leaves of *A. muricata* in a 1:1 chloroform/methanol mixture caused 67% inhibition of *P. falciparum* [8]. Moreover, hexane, ethyl acetate and methanol extracts of *A. muricata* leaf exhibited moderate activities against chloroquine sensitive but not chloroquine resistant strains of *P. falciparum*. However, in their study the most potent ethyl acetate extract was toxic to human monocytes. In a similar approach, *A. muricata* leaf powder (from Malaysia) defatted with hexane and sequentially extracted with dichloromethane, methanol and water showed promising activity against *P. falciparum*, with negligible toxicity against bovine cells [9]. In another study, *A. muricata* leaf aqueous, ethanol and pentane extracts showed moderate antiplasmodial activity [10]. Few results have been reported for the antiplasmodial activity of isolated constituents from *A. muricata*, mainly acetogenins [10]. Anonaine isolated from the fruit of *A. muricata* showed antiplasmodial activity [32] with low cytotoxicity.

Moderate antiplasmodial activity of gallic acid was reported [33], and authors concluded that the activity was linked to its very strong antioxidant capacity [33]. In our study, β-sitosterol-3-*O*-β-d-glucopyranoside showed no activity, contrary to the reports of other authors who claimed appreciable potency for the same compound isolated from the bark of *Dacryodes edulis* (Burseraceae) [34].

Also, extracts and fractions from leaf, twig, root, stem bark and fruit of *A. reticulata* showed activities against *P. falciparum.* As far as we know, this is the first report on the antiplasmodial activity of extracts from *A. reticulata*. However, *A. reticulata* has been investigated for activity against other protozoan parasites. The *in vitro* anti-leishmanial activity of extracts from the leaf and seed of the plant was studied [35], and results showed that the oxoaporphine alkaloid liriodenine isolated from the leaf dichloromethane extract was active against promastigote forms of *L. amazonensis*, *L. braziliensis* and *L. guyanensis* and against the intracellular amastigote forms of *L. amazonensis* [35].

## 4. Conclusions

Our results highlight the antiplasmodial activity and lack of cytotoxicity of extracts from *A. muricata* and *A. reticulata*. Detailed studies are ongoing to characterize the active principles and further structure-activity-relationships.

## References

[1] World Health Organization (2014). Malaria Report.

[2] World Health Organization (2010). World Health Organization Treatment Recommendation.

[3] Dondorp A.M., Nostern F., Yi P., Das D., Phyo A.P., Tarning J., Lwin K.M., Ariey F., Hanpithakpong W., Lee S.J. (2009). Artemisinin-Resistant *Plasmodium falciparum* Malaria. N. Engl. J. Med..

[4] Meshnick S.R., Taylor T.E., Kamchonwongpaisan S. (1996). Artemisinin and the antimalarial endoperoxides: from herbal remedy to targeted chemotherapy. Microbiol. Rev..

[5] Tsabang N., Tsouh F.P.V., Yamthe T.L.R., Noguem B., Bakarnga-Via I., Dongmo N.M.S., Boyom F.F. (2012). Ethnopharmacological survey of Annonaceae medicinal plants used to treat malaria in four areas of Cameroon. J. Ethnopharmacol..

[6] Pinto A.C., Andrade S.R., Ferreira F.R., Kinpara D.I. (2005). Annona species. International Center for under Utilised Crops.

[7] Boyom F.F., Tsouh F.P.V., Tchokouaha Y.L.R., Ngoutane M.A., Madiesse K.A.E., Mbacham F.W., Tsamo E., Amvam Z.P.H., Jiri G., Rosenthal P.J. (2011). Potent antiplasmodial extracts from Cameroonian Annonaceae. J. Ethnopharmacol..

[8] Bidla G., Titanji V.P.K., Joko B., Ghazali G.E., Bolad A., Berzins K. (2004). Antiplasmodial activity of seven plants used in african folk medicine. Indian J. Pharmacol..

[9] Razak M.A., Adlin A., Rosnani A.N., Mohd I.W., Siti H.S., Noor R.A., Zakiah I. (2014). Effect of selected local medicinal plants on the asexual blood stage of chloroquine resistant *Plasmodium falciparum*. BMC Comp. Altern. Med..

[10] Menan H., Banzouzi J.T., Hocquette A., Pelissier Y.Y., Blache Y., Kone M., Mallie M., Ake A.L., Valentin A. (2006). Antiplasmodial activity and cytotoxicity of plants used in West African traditional medicine for the treatment of malaria. J. Ethnopharmacol..

[11] Chavan S.S., Shamkuwar P.B., Damale M.G., Pawar D.P. (2014). A comprehensive review on *Annona reticulata*. Int. J. Pharm. Sc. Res..

[12] Alali F.Q., Liu X.X., McLaughlin J.L. (1999). Annonaceous acetogenins: Recent progress. J. Nat. Prod..

[13] Cedillo-Rivera R., Ramfrez A., Munoz O. (1992). A rapid colorimetric assay with the Tetrazolium salt MTT and Phenazine Methosulfate (PMS) for viability of *Entamoeba histolytica*. Arch. Med. Res..

[14] Boyom F.F., Madiesse K.E., Tepongning R., Ngouana V., Mbacham W.F., Tsamo E., Amvam Zollo P.H., Gut J., Rosenthal P.J. (2009). Antiplasmodial activity of extracts from seven medicinal plants used in malaria treatment in Cameroon. J. Ethnopharmacol..

[15] Boyom F.F., Tsouh F.P.V., Tchokouaha Y.L.R., Spangenberg T., Mfopa N.A., Kouipou T.R.M., Mbouna J.C., Donkeng D.F.V., Zollo A.P.H. (2014). Repurposing the open access malaria box to discovery potent inhibitors of *Toxoplasma gondii* and *Entamoeba histolytica*. Antimicrob. Agents Chemother..

[16] Lambros C., Vanderberg J.P. (1979). Synchronization of *Plasmodium falciparum* erythrocytic stages in culture. J. Parasitol..

[17] Singh A., Rosenthal P.J. (2001). Comparison of efficacies of cysteine protease inhibitors against five strains of *Plasmodium falciparum*. Antimicrob. Agents Chemother..

[18] Wright H.E., Burton W., Berry R.C. (1962). Identification of stigmasteryl d-glucoiside in aged burley tobacco. J. Org. Chem..

[19] Mba’ning B.M. (2012). Etude Phytochimique et Pharmacologique de SalaciacamerunensisLoes., Salaciadimidia Hall. (Celastraceae) et Crinumnatans Baker (Amaryllidaceae). Ph.D. Thesis.

[20] Ruben F.G., Keith S.B. (1976). Alkaloids of three *Aspidosperma* species. Phytochemistry.

[21] Buitrago D.A., Rojas V.J., Cote V., Bruno-Colmenárez J., de Delgado G.D. (2010). NMR elucidation and crystal structure analysis of 1-hydroxy-3,6-dimethoxy-8-methyl-9*h*-xanthen-9-one (lichexanthone) isolated from *Vismia baccifera* (Guttiferae). Bol. Lat. Am. Caribe Plantas Med. Aromát..

[22] Eldahshan O.A. (2010). Isolation and structure elucidation of phenolic compounds of Carob leaves grown in Egypt. Curr. Res. J. Biol. Sci..

[23] Zhang H.M., Wang C.F., Shen S.M., Wang G.L., Liu Z.M., Wang Y.Y., Du S.S., Liu Z.L., Liu P., Deng Z.W. (2012). Antioxidant phenolic compounds from Pu-erh tea. Molecules.

[24] Ramiarantsoa H., Koffi B.A., Kouamé M.A., Djakouré L.A. (2008). Le *O*-β-d-glucoside du β-sitostérol Isolé des feuilles de *Ravenala madagascariensis*. J. Soc. Ouest-Afr. Chim..

[25] Faizi S., Khan R.A., Azher S., Khan S.A., Tauseef S., Ahmad A. (2003). New antimicrobial alkaloids from the roots of *Polyalthia longifolia* var pendula. Planta Medica.

[26] Arriaga A.M., Feitosa E.M., Lemos T.L., Santiago G.M., Lima J.Q., de Oliveira M.C., Vasconcelos J.N., Rodrigues F.E., Gomes T.B., Braz-Filho R. (2008). Chemical constituents and insecticidal activity of *Rollina leptopetala*. Nat. Prod. Commun..

[27] Costa E.V., Assis M.F., Lucia B.M.P., Broga R.M., Delarmelina C., Duarte T.C.M., Ruiz T.G.L.A., Carvalho J.E., Maia B.H. (2011). Chemical constituents isolated from the bark of *Guatteria blepharophylla* (Annonaceae) and their antiproliferative and antimicrobial activities. J. Braz. Chem. Soc..

[28] Sampath M. (2013). Isolation and identification of gallic acid from *Polyalthia longifolia* (Sonn.) Thawaites. Int. J. Pharm. Biol. Sci..

[29] Dinesh K.Y., Neetu S., Shaima R., Mahendra S., Gautam P., Rakesh M. (2011). Anti-ulcer constituents of *Annona squamosa* twigs. Fitoterapia.

[30] Nwaka S., Ramirez B., Reto B., Maes L., Douglas F., Ridley R. (2009). Advancing drug innovation for negleted diseases. Critera for lead progression. PLoS Negl. Trop. Dis..

[31] Weniger B., Robledo S., Arango G.J., Deharo E., Aragon R., Munoz V., Callapa J., Lobstein A., Anton R. (2001). Antiprotozoal activities of Colombian plants. J. Ethnopharmacol..

[32] Graziose R., Rathinasabapathy T., Lategan C., Poulev A., Smith P.J., Grace M., Lila M.A., Raskin I. (2011). Antiplasmodial activity of aporphine alkaloids and sesquiterpene lactones from *Liriodendron tulipifera* L.. J. Ethnopharmacol..

[33] Ndjonka D., Bärbel B., Agyare C., Zimbres F.M., Lüersen K., Hensel A., Carsten W., Liebau E. (2012). *In vitro* activity of extracts and isolated polyphenols from West African medicinal plants against *Plasmodium falciparum*. Parasitol. Res..

[34] Zofou D., Tematio E.L., Ntie-Kang F., Tene M., Ngemenya M.N., Tane P., Titanji V.P.K. (2013). New Antimalarial Hits from *Dacryodes edulis* (Burseraceae)—Part I: Isolation, *In Vitro* Activity, *In Silico* “drug-likeness” and Pharmacokinetic Profiles. PLoS One.

[35] De Lima J.P.S., Pinheiro M.L.B., Santos A.M.G., Pereira J.L.S., Santos D.M.F., Barison A., Silva-Jardim I., Costa E.V. (2012). *In Vitro* Atileishmanial and Cytotoxic Activities of *Annona mucosa* (Annonaceae). Rev. Virtual Quim..

